# Macro–Micro Aging Mechanisms and Correlation Analysis of Asphalt Under Light–Heat–Oxygen Coupling Action

**DOI:** 10.3390/ma19142966

**Published:** 2026-07-09

**Authors:** Han Xi, Shixiong Hu, Yuanxun Zheng, Senfeng Fu

**Affiliations:** 1 School of Smart Transportation and Intelligent Construction Engineering, Huanghe Jiaotong University, Jiaozuo 454950, China; shu@esu.edu; 2 Institute of Smart Transportation and New Quality Productivity, Huanghe Jiaotong University, Jiaozuo 454950, China; 3 College of Water Resources and Environment, Zhengzhou University, Zhengzhou 450001, China; yxzheng@zzu.edu.cn; 4 Henan Haide Transportation Technology Co., Ltd., Zhengzhou 450046, China; zziafu@163.com

**Keywords:** road engineering, light–heat–oxygen aging, macroscopic properties, microscopic mechanism, correlation

## Abstract

To investigate the macroscopic and microscopic aging mechanisms of asphalt under combined light, heat, and oxygen exposure in southwestern China, four aging schemes were designed and tested on three base asphalts. Chemical changes—components, elements, and functional groups—were characterized by SARA and elemental analyses, and FTIR. High- and low-temperature rheological properties were measured via MSCR and BBR tests, and the gray correlation method linked microscopic mechanisms to macroscopic performance. The results show that coupled aging causes bond breakage and dehydrogenation, with recombined small molecules increasing the asphaltene content. Dehydrogenated bonds combine with oxygen to form polar functional groups, increasing chain stiffness and restricting internal rotation. Macroscopically, high-temperature performance improves while low-temperature performance degrades. Carbonyl and sulfoxide indices serve as effective aging indicators. Correlation analysis identifies functional group changes as the most significant factor affecting rheological decay. Engineering implications are as follows: oxygen-containing polar groups enhance chemical stability but accelerate low-temperature performance loss; thus, their evolution should be monitored in high-radiation, high-temperature regions to mitigate premature cracking.

## 1. Introduction

With China’s road transportation development and the Belt and Road Initiative, the southwest region—characterized by mountains, plateaus, low latitudes, and high altitudes—experiences strong UV, high temperatures, and thin oxygen, which severely accelerate asphalt pavement aging and shorten its service life [1,2,3]. Studying asphalt aging under such coupled environmental conditions is therefore critical.

Extensive research has addressed asphalt aging under single factors. Camargo et al. [4] and Zhu et al. [5,6] focused on thermal oxygen aging, reporting that oxygen plays a major role in hardening, causing significant rutting factor changes. Hu et al. [7] found that thermal oxidation reduces light components and increases heavy fractions. Regarding UV-dominated aging, Pandhawale et al. [8], He et al. [9], Gao [10], and Li [11] demonstrated UV-induced chain scission, surface porosity, and rheological deterioration, with UV causing greater physical degradation than that of the freeze–thaw cycle. However, these studies isolated thermal oxygen or UV effects without systematic coupling of light, heat, and oxygen—a combination especially relevant to high-altitude southwest China. Moreover, quantitative links between chemical composition (functional groups, elements, and components) and rheological decay are scarce, and the decisive environmental factor remains unidentified.

To address these gaps, this study designed four coupled aging schemes, including an anaerobic condition (0% O_2_), measuring three chemical dimensions and four rheological parameters on three base asphalts, and applied gray correlation analysis to quantify the structure–activity relationships. As such, we aim to (1) reveal the macroscopic and microscopic aging mechanisms under coupled light–heat–oxygen; (2) analyze rheological decay patterns in relation to chemical changes; and (3) identify key microscopic factors governing aging via gray correlation. Preliminary findings demonstrated that functional group indices correlate strongly (0.74–0.89) with rheological properties, with the carbonyl index being an early indicator and the sulfoxide index reflecting anti-aging capacity. This work advances from single-factor to coupled aging, from separate to integrated structure–activity relationships, and provides a microscopic theoretical basis and data support for developing anti-aging asphalt materials tailored to southwest China’s environment.

## 2. Materials and Test Methods

### 2.1. Asphalt Material

Three different types of 70# base asphalts, namely PetroChina Asphalt (ZSY), HSBC Asphalt (HF), and Shell Asphalt (K), were selected as the research objects. The basic physical properties were determined in accordance with the “Technical Specifications for Construction of Highway Asphalt Pavement” (JTG F40-2017) and “Test Procedures for Asphalt and Asphalt Mixture in Highway Engineering” (JTG E20-2011), and the results are shown in Table 1. From Table 1, it can be seen that these three types of asphalt, respectively, represent the typical 70# base asphalt with different crude oil sources and production processes in the Chinese market. They have been widely used in road engineering in the southwestern region and various climate zones. Therefore, the results of this study have good engineering representativeness.

Table 1 specifies the technical requirements in accordance with JTG F40-2017, and the test methods are based on JTG E20-2011.

It should be noted that the elongation values of the ZSY and K asphalts at 15 °C (16.35 cm and 23.40 cm, respectively) are below the technical requirement of ≥100 cm specified in JTG F40-2017 for 70# base asphalt. This is because these two asphalts are sourced from specific crude oils and produced by processes that prioritize high-temperature stability over low-temperature ductility. Nonetheless, these asphalts are still used in certain road engineering contexts in southwestern China, especially in regions where low-temperature cracking is not the primary issue. Moreover, the objective of this study is to compare the aging mechanisms and structure–activity relationships across different asphalt types rather than to evaluate compliance with specification limits. The significant difference in elongation among the three asphalts actually provides a broader basis for examining how initial physical properties affect aging behavior. Therefore, the use of these asphalts is justified for research purposes.

### 2.2. Light–Heat–Oxygen Coupling Aging Test

**Figure 1:**
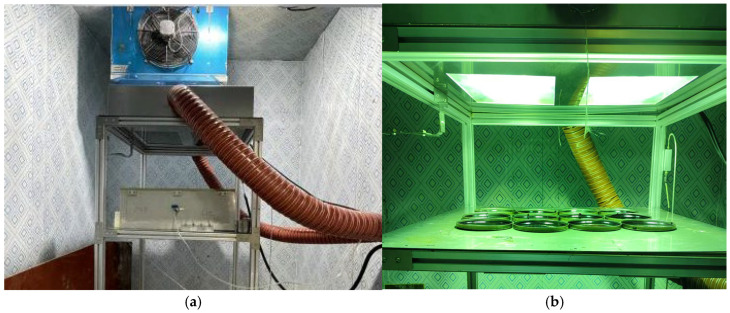
Multi-functional environmental box and the asphalt samples placed within it underwent aging: (**a**) Multi-functional environmental box; (**b**) the asphalt samples placed within it that underwent aging.

The self-developed multi-functional environmental box, as shown in Figure 1, was adopted to conduct 24 h continuous aging of the selected asphalt. In response to the influence of the light–heat–oxygen coupling effect on asphalt aging, a multi-factor control scheme was adopted in the experiment: To simulate the ultraviolet light intensities in different regions, the selected ultraviolet radiation intensities were 65 W/m^2^ and 85 W/m^2^, respectively, simulating three and four times the average irradiance intensity in the southwest region, as shown in Table 2. Since asphalt only undergoes ultraviolet aging below 40 °C rather than thermal oxygen aging, and considering that the surface temperature of asphalt pavement can reach 60 °C in summer [12], the test temperatures selected are 40 °C and 60 °C. The temperature set in this experiment is the air temperature inside the environmental chamber. The 60 °C temperature was selected based on the actual achievable temperature of the asphalt pavement surface in summer in order to simulate the real thermal state of the asphalt under operational conditions. The oxygen content was set at two states: normal oxygen (20%) and anaerobic (0%). The light–thermal–oxygen coupling aging test scheme is shown in Table 3. The aging time was set at 0, 1, 2, 4, and 8 days. Based on the actual ultraviolet radiation total amount in the southwest region over a half-year period (average intensity 21.51 W/m^2^ × monthly irradiation duration 116 h × 6 months), which is equal to the total energy of continuous irradiation of 65 W/m^2^ for 8 days in the laboratory, and considering that the increment of carbonyl/sulfone groups after half a year of field exposure tests was consistent with the laboratory results after 8 days of irradiation, it was determined that 8 days is equivalent to half a year. The thickness of the asphalt sample in this test is 1.5 mm. Based on the 14 cm diameter disk used in the test and the calculation of asphalt density, it was established that approximately 23 g of asphalt should be poured into each aging disk, and after pouring, the sample should be heated to ensure a uniform distribution of the asphalt within the aging disk. Based on the typical climatic characteristics of the southwestern region and the results of the pre-experiment, the above four sets of schemes were selected to separately examine the main effects of ultraviolet intensity, temperature, and oxygen, as well as their coupling effects. The aging effects of the unmentioned combinations can be inferred from the comparisons of the existing combinations. This accelerated aging cycle corresponds to 1.5 years in the field, mainly targeting the initial to middle stages of aging sensitivity; the longer-term aging behavior and its coupling with factors such as load will be investigated in subsequent studies.

### 2.3. Chemical Composition Test and Its Index Calculation

#### 2.3.1. Chemical Composition Test

The determination of chemical composition under coupled aging mainly includes component composition, elemental composition, and chemical functional group composition. Four-component and elemental analysis experiments were conducted, respectively, using a rod-shaped thin-layer chromatograph and an organic elemental analyzer to determine the changes in composition, including elemental composition, of asphalt. Among them, the oxygen element in asphalt could not be measured through experiments but was obtained through the difference method. The composition changes in asphalt functional groups were determined by an infrared spectrometer. The test range of the infrared spectrometer was 400–4000 cm^−1^, the resolution was 4 cm^−1^, and the number of scans was 64.

#### 2.3.2. The Component Content and Colloidal Instability Index Calculation

Asphalt can be divided into saturate (*S*) and aromatic (*A_s_*) light components and resin (*R*) and asphaltene (*A_r_*) recombination components. The colloidal instability index (*I_c_*) represents the change in the colloidal structure of asphalt. The *I_c_* can be obtained by calculating the proportion of asphalt recombination components and light components, as shown in Equation (1) [13].

(1) $$I_{C}=\frac{A_{s}+S}{A_{r}+R}$$

where *I_c_* is the colloidal instability index, *A_r_* is the aromatic, *R* is the resin, *A_s_* is the asphaltene, and *S* is the saturated.

#### 2.3.3. The Hydrogen-to-Carbon Ratio Calculation

The hydrogen-to-carbon ratio refers to the ratio of hydrogen content to carbon content in asphalt. During the aging process of asphalt, the lower the hydrogen–carbon ratio, the stronger the dehydrogenation reaction of asphalt, and the calculation method is shown as Equation (2) [14].

(2) $$H/C=\frac{N(H)}{N(C)}$$

#### 2.3.4. The Sulfoxide and Carbonyl Functional Group Indices Calculation

According to the Lambert–Beer law, the characteristic peak area at 2000~6000 cm^−1^ was taken as a reference value [15]. Equations (3) and (4) are used to calculate the sulfoxide group index (*SI*) and carbonyl group index (*CI*).

(3) $$SI=\frac{A_{S=O}}{A_{2000-6000}}$$

(4) $$CI=\frac{A_{C=O}}{A_{2000-6000}}$$

where *SI* is the sulfoxide index; *A_S=O_* is the 1030 cm^−1^ characteristic peak area; *CI* is the carbon index; *A_C=O_* is the 1700 cm^−1^ characteristic peak area; and *A*_2000–6000_ is the 2000~6000 cm^−1^ band total area.

### 2.4. Rheological Performance Test and Its Index Calculation

#### 2.4.1. Rheological Performance Test

The multiple stress creep recovery test (MSCR) and the bending beam rheometer (BBR) were conducted using a dynamic shear rheometer and a bending beam rheometer, respectively, to determine the high- and low-temperature rheological properties of asphalt under the action of a coupled environment. The MSCR temperature was selected as 64 °C, and continuous loading tests were conducted using two stresses, 0.1 kPa and 3.2 kPa, respectively. The BBR temperature was selected as −12 °C.

#### 2.4.2. Calculation of Non-Recoverable Creep Modulus and Creep Recovery Rate

The high-temperature creep performance indicators of asphalt obtained from the MSCR include the mean non-recoverable creep flexibility (*J_nr_*) and the mean creep recovery rate (*R*). Among them, Jnr represents the creep relaxation deformation of asphalt under stress load, and R represents the resilience of asphalt under stress load [16]. The calculation methods of Jnr and R are shown in Equations (5) and (6).

(5) $$J_{nr}=\frac{\varepsilon_{u}}{\delta }\times 100$$

(6) $$R=\frac{\varepsilon_{p}-\varepsilon_{u}}{\varepsilon_{p}}\times 100$$

where *J_nr_* is the irrecoverable creep modulus; *R* is the creep recovery rate; *ε_u_* is the unrecovered strain; *ε_p_* is the initial strain; and *δ* is the loading stress, which is taken as 0.1 kPa and 3.2 kPa, respectively.

#### 2.4.3. Calculation of Creep Stiffness Modulus and Creep Rate

The evaluation indicators of the low-temperature creep performance of asphalt mainly include the creep stiffness modulus (*S*) and creep rate (*m*); the calculation methods are shown in formulas (7) and (8) [17]. According to the requirements of Superpave, the *S* value of the asphalt after the test shall not be less than 300 MPa, and the *m* value shall not be less than 0.3.

(7) $$S=\frac{Pl^{3}}{4bh^{3}v(t)}$$

(8) $$m=\frac{d\left[\log S(t)\right]}{d\left[\log(t)\right]}$$

where *S* is the creep stiffness modulus; *m* is the creep rate; *v(t)* is the deflection of the curved beam (mm); *p* is the central load (N); *b* is the width of the beam (mm); *h* is the beam height (mm); and *l* is the beam span (mm).

### 2.5. Relevance Calculation

The correlation degree is calculated by using mathematical software based on the gray correlation method, and its internal calculation logic is shown as follows [18,19]:

(1) Reference series and comparative series

The reference sequence is composed of data that can represent the behavior characteristics of the system, and the comparison sequence is composed of data that impacts the reference sequence.

(9) $$X_{0}=\{X(1),X(2),X(3),\ldots ,X(n)\}$$

(10) $$X_{i}(n)=\{X_{i}(1),X_{i}(2),X_{i}(3),\ldots ,X_{i}(n)\},i=1,2,\ldots ,n$$

This paper uses the rheological property index and chemical composition index as reference series, and the molecular weight and molecular structure index as comparative series.

(2) Correlation degree calculation

The samples must be dimensionless since the dimensions of different data samples are different to ensure the equivalence and homogeneity of each factor:

(11) $$X_{i}^{\prime }=\{\frac{X(1)}{X(1)},\frac{X(2)}{X(1)},\frac{X(3)}{X(1)},\ldots ,\frac{X(n)}{X(1)}\}$$

(12) $$X_{i}^{\prime }(n)=\{\frac{X_{i}(1)}{X_{i}(1)},\frac{X_{i}(2)}{X_{i}(1)},\frac{X_{i}(3)}{X_{i}(1)},\ldots ,\frac{X_{i}(n)}{X_{i}(1)}\},i=1,2,\ldots ,n$$

(3) Calculate the absolute difference:

(13) $$X_{0}^{0^{‘}}(n)=\{X_{0}^{0^{\prime }}(1)-X_{0}^{0^{\prime }}(1),X_{0}^{0^{\prime }}(2)-X_{0}^{0^{\prime }}(1),\ldots ,X_{0}^{0^{\prime }}(n)-X_{0}^{0^{\prime }}(1)\}$$

(14) $$X_{i}^{0^{‘}}(k)=\{X_{i}^{0^{\prime }}(1)-X_{i}^{0^{\prime }}(1),X_{i}^{0^{\prime }}(2)-X_{i}^{0^{\prime }}(1),\ldots ,X_{i}^{0^{\prime }}(n)-X_{i}^{0^{\prime }}(1)\}$$

(4) Calculate the correlation coefficient:

(15) $$\left|s_{0}^{\prime }\right|=\left|\sum_{m=2}^{n-1}X_{0}^{0^{\prime }}(m)+\frac{1}{2}X_{0}^{0^{\prime }}(n)\right|$$

(16) $$\left|s_{i}^{\prime }\right|=\left|\sum_{m=2}^{n-1}X_{i}^{0^{\prime }}(m)+\frac{1}{2}X_{i}^{0^{\prime }}(n)\right|$$

(17) $$\left|s_{i}^{\prime }-s_{0}^{\prime }\right|=\left|\sum_{m=2}^{n-1}\left(X_{i}^{0^{\prime }}(m)-X_{0}^{0^{\prime }}(m))+\frac{1}{2}(X_{i}^{0^{\prime }}(n)-X_{0}^{0^{\prime }}(n)\right)\right|$$

(5) Calculate the relative correlation degree:

(18) $$r_{0i}^{\prime }=\frac{1+\left|s_{0}^{\prime }\right|+\left|s_{i}^{\prime }\right|}{1+\left|s_{0}^{\prime }\right|+\left|s_{i}^{\prime }\right|+\left|s_{i-0}^{\prime }\right|}$$

According to the calculated results of the relative correlation degree $r_{0i}^{\prime }$, the larger the value, the better the correlation between the comparison sequence and the reference sequence.

Some studies have suggested that a correlation between calculation indicators can be demonstrated when the correlation coefficient is ≥0.6 [20,21]. Therefore, in order to ensure a significant correlation among the calculated indicators, we adopted a correlation coefficient of 0.7 as the threshold to analyze the chemical composition and rheological properties of asphalt.

### 2.6. Experimental Method

**Figure 2:**
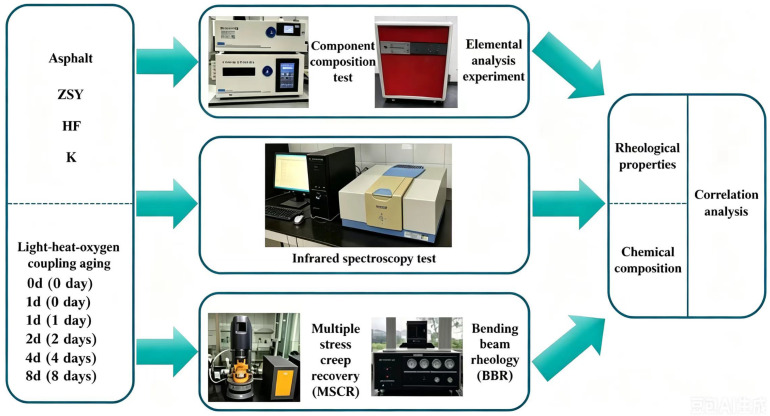
Diagram of experiment method.

To explore the influence of light–heat–oxygen coupling aging on the chemical composition and rheological properties of asphalt, four test schemes were selected in this paper on three commonly used asphalts in engineering at 0 d, 1 d, 2 d, 4 d, and 8 d. The component, elemental, and functional group composition mechanisms of the samples were determined through tests such as four-component elemental analysis and infrared spectroscopy. The high- and low-temperature properties of asphalt were measured through multiple stress creep and bending beam rheological tests. Using gray correlation method calculations, the correlation between the chemical composition of asphalt and its rheological properties was established, revealing the chemical formation mechanism behind rheological property decay. The specific experimental scheme is shown in Figure 2.

## 3. Results and Analysis

### 3.1. Chemical Composition Index

#### 3.1.1. The Change in Asphalt Component Composition and I_C_

**Figure 3:**
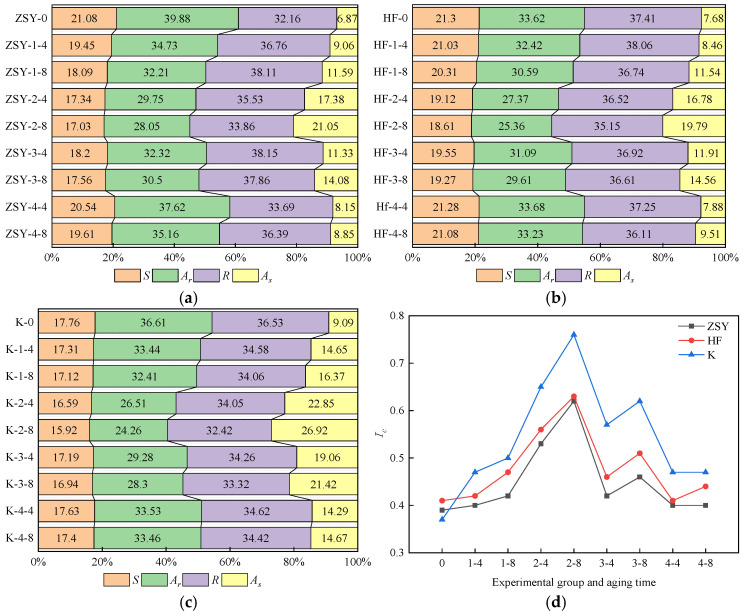
Composition and *I_c_* change in asphalt: (**a**) ZSY component composition; (**b**) HF component composition; (**c**) K component composition; (**d**) the change in *I_c_*.

The component composition tests and colloid instability coefficient calculation results of different asphalts after UV aging for 0, 4, and 8 days are shown in Figure 3.

As can be seen from Figure 3, with the increase in aging time, the aromatic content of asphalt decreases significantly, the asphaltene content increases significantly, while the changes in the content of resin and saturated content are relatively small. The reason is that the main component of aromatic hydrocarbons is aromatic hydrocarbons. Under the action of oxygen, aromatic hydrocarbons undergo oxidative dehydrogenation reactions. At the same time, since the molecular structure of aromatic hydrocarbons mainly exists in the form of long-chain molecules, the structure itself has relatively weak stability. Therefore, under high molecular energy and high temperatures, long-chain molecules of aromatic hydrocarbons undergo carbon-hydrogen bond-breaking reactions, forming free small molecules. This leads to a reduction in the aromatic content in asphalt. Asphaltene is mainly composed of aromatic ring molecules, existing in a cyclic structure. Due to the inability of ordinary light and temperature energy to open the ring, the chemical properties of the aromatic ring molecules in asphalt are extremely stable. Therefore, the free small molecules formed after the fracture of aromatic hydrocarbons are connected to the large molecules of the aromatic ring under a condensation reaction produced by ultraviolet light and high temperature, thereby increasing the content of asphaltene. The saturated components in asphalt are mainly composed of saturated hydrocarbons existing in the form of carbon–carbon single bonds with strong stability, and thus, are less affected by aging. The change in resins mainly depends on the difference in the rate of transformation from aromatic components to resins and from resins to asphaltenes.

By comparing the changes in *A_s_* and *A_r_* of asphalt in different experimental groups, it can be found that the asphalt component change in experimental group 2 was the most significant among the four experimental groups, indicating that under the combined aging effect of ultraviolet light and high temperature, the degree of asphalt aging is relatively large. The reason is that under the combined aging conditions of ultraviolet and high temperature, the chemical bonds in asphalt are simultaneously subjected to the dual destruction and condensation effects of ultraviolet photon energy and high-temperature thermal decomposition, resulting in a greater reduction in aromatic components and an increase in asphaltenes. In addition, high temperatures enhance the activity of free small molecules, enabling aromatic rings to capture more small molecules and form larger and more stable cyclic structures. In Test 4, the asphalt component changed the least, indicating that the aging process of asphalt will slow down without the participation of oxygen. The reason is that although the long-chain molecules of asphalt will still break in an oxygen-free environment, due to the lack of oxygen participation, the dehydrogenation reaction of asphalt is difficult to complete. Therefore, fewer free small molecules were available for the connection of aromatic rings, and thus the changes in aromatic components and asphaltenes were relatively small.

It can be seen from Figure 3 that the *I_c_* value of asphalt increases after aging under different environmental conditions, indicating that the colloid structure of asphalt gradually changes from a sol-type structure to a gel structure after aging. Among them, the Ic value of test group 2 after aging for 8 days is the largest, indicating that the asphalt colloid at this time is closest to the gel structure. From the changes in components, it can be known that at this time, under the dual bonding and condensation effects of strong photon energy and high temperature, the content of polar and unstable components in asphalt is the smallest, and the content of stable macromolecular structure components is the largest. Therefore, the chemical stability of asphalt is the strongest. It can be seen from the figure that the Ic value of K asphalt is significantly higher than that of the other two types of asphalt, indicating that K asphalt is closer to the gel structure.

#### 3.1.2. The Change in Asphalt Elemental Composition and H/C

**Figure 4:**
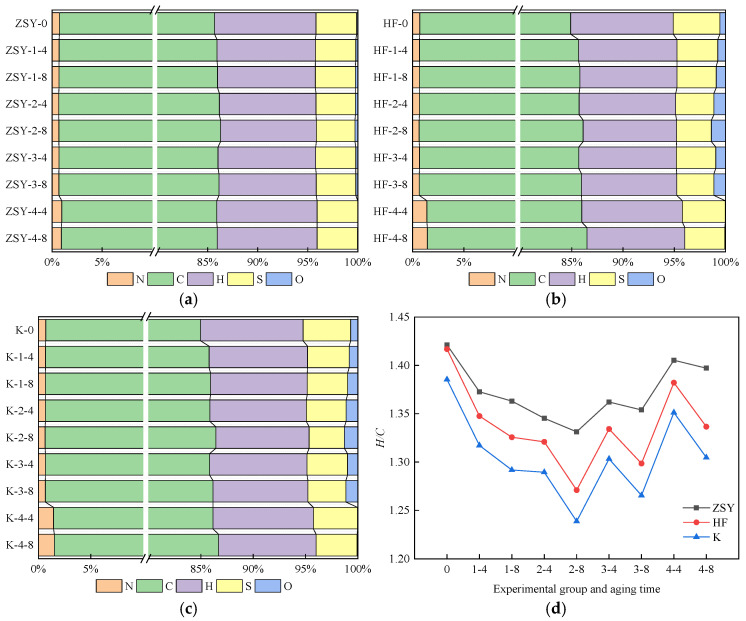
Elemental composition and H/C change in asphalt: (**a**) ZSY elemental composition; (**b**) HF elemental composition; (**c**) K elemental composition; (**d**) the change in H/C.

The elemental composition and *H/C* changes in asphalt aging for 0, 4, and 8 days under the coupling effect of light, heat, and oxygen are shown in Figure 4 and Table 4.

As can be seen from Figure 4, with the increase in aging time and degree, the C element in asphalt increases and the H element decreases, indicating that a dehydrogenation reaction occurs in asphalt during the aging process. Under the action of light, heat, and oxygen, after asphalt ages, a carbon–hydrogen bond-breaking reaction occurs. The hydrogen atoms after breaking will detach and undergo oxidation reactions under the action of oxygen, forming oxygen-containing macromolecules and polar components with larger volumes, thereby increasing the chemical stability of asphalt. The carbon elements in the fracture will undergo aggregation reactions, gradually aggregating from free carbon to solid carbon and eventually becoming fixed in the form of asphaltenes, further enhancing the chemical stability of the asphalt. The increase in the O element indicates that under the aging effect, oxygen atoms will combine with other elements inside the asphalt to form polar functional groups such as -COOH, S=O, and C=O. Due to the existence of a permanent dipole, polar functional groups will generate electrostatic forces, increasing the friction between different molecules of aged asphalt. After oxygen enters the interior of asphalt, it will connect to the side groups of the molecular chain due to permanent dipolarity. At the same time, under the interaction of polar groups, the stiffness of the asphalt molecular chain increases, making rotation difficult. This indicates that oxygen plays a decisive role in the aging of asphalt; that is, the essence of asphalt aging is oxidation.

The *H/C* of asphalt decreases after aging, indicating that the cycloalkanes and aromatic hydrocarbons inside the asphalt decrease after aging. This is because, under the catalysis of ultraviolet light, cycloalkanes and aromatic hydrocarbons undergo molecular side-chain breakage and dehydrogenation reactions. Among them, cycloalkanes generate cycloolefins after dehydrogenation, and aromatic hydrocarbons generate aromatic rings after dehydrogenation. Since both cycloolefins and aromatic rings are cyclic molecules with relatively high stiffness, they have extremely high chemical stability. Meanwhile, compared with cycloolefins, aromatic rings not only have higher stability but are also the main molecules constituting asphaltenes. Therefore, the decrease in *H/C* of asphalt after aging is more affected by aromatic hydrocarbons. It can be seen from Figure 4d that the *H/C* ratio of ZSY is higher than that of the other two types of asphalt, indicating that the anti-aging performance of ZSY is better.

#### 3.1.3. The Change in Functional Groups Composition

**Figure 5:**
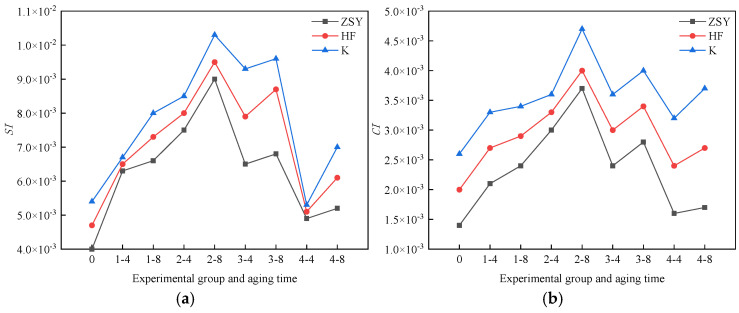
*SI* and *CI* change in asphalt: (**a**) *SI* change in asphalt; (**b**) *CI* change in asphalt.

The calculation results of the sulfoxide and carbonyl indices are shown in Figure 5.

As can be seen from Figure 5, the *CI* and *SI* of asphalt in different test groups all show an increasing trend with an increase in aging time. This is because during the aging process of asphalt, due to the effect of the chain reaction of free radicals, the active groups in the asphalt molecules decompose to generate free radicals. These free radicals react with oxygen in the air and further transform into components containing carbonyl and sulfoxide functional groups. This increases the content of carbonyl and sulfoxide groups. At the same time, it can be seen from the increase in CI and *SI* at 4 and 8 days of aging that growth is relatively fast in the early stage of aging but slows down in the later stage. This is because in the early stage of aging, asphalt rapidly undergoes bond-breaking reactions under ultraviolet light; the molecular structure of aromatic components is destroyed, accompanied by dehydrogenation reactions, thereby resulting in a large number of unsaturated hydrocarbons and polar double-bond molecules. This leads to a rapid increase in the number of carbonyl and sulfoxide functional groups. In the later stage of aging, the aromatic components inside the asphalt are basically transformed into resins and asphaltenes, and the bond-breaking and dehydrogenation reactions decrease. At the same time, due to the gradual transformation of carbon atoms in the asphalt into solid carbon and the formation of aromatic rings connected to the molecular main chain, as well as the reduction in active sulfur atoms inside the asphalt, the available carbon and sulfur atoms for reactions also gradually decrease. This further leads to a gradual slowdown in the growth of carbonyl and sulfoxide functional groups.

By comparing the different experimental groups in Figure 5, it can be seen that the *CI* and *SI* values under the combined action of ultraviolet irradiation and high temperature in experimental group 2 are the largest, indicating that the contents of carbonyl and sulfoxide functional groups are the highest at this time. The reason is that under ultraviolet irradiation, asphalt molecules generate hydroperoxides under the action of oxygen and excitation, which, in turn, produce polar functional groups of carbonyl and sulfoxide groups. At the same time, ultraviolet light also triggers chemical reactions such as isomerization, dealkylation, and cracking, further accelerating the aging process of asphalt. High temperatures accelerate the oxidation process of asphalt, resulting in an increase in asphaltene content and changes in the colloid structure. In the anaerobic condition of test group 4, the values of *CI* and *SI* were the smallest. This is because there was a lack of sufficient oxygen. After the asphalt molecules broke, they could not form oxygen-containing functional groups such as S=O and C=O with oxygen atoms. Therefore, *CI* and *SI* were relatively small.

By comparing *CI* and *SI* in Figure 5, it can be seen that the absolute value of SI is much greater than that of *CI* in each asphalt, but the growth rate of *CI* within the same aging period is faster than that of SI. This observation is consistent with known reaction pathways: the free radical intermediates formed during asphalt aging can readily combine with oxygen to produce carbonyl groups (C=O), whereas the formation of sulfoxide groups (S=O) requires the presence of sulfur atoms and proceeds via a different oxidation mechanism that is often slower [22]. Therefore, the carbonyl index (*CI*) is considered a sensitive early indicator of aging initiation, while the sulfoxide index (*SI*), due to its higher absolute value and slower formation kinetics, reflects the cumulative anti-aging capacity of the asphalt and its ability to resist further oxidative functionalization. This interpretation is supported by the gray correlation results in Section 3.3, where *CI* and *SI* both show strong correlations with rheological decay but with slightly different weighting.

### 3.2. Rheology Index

#### 3.2.1. The Change in High-Temperature Rheological Properties Index

**Figure 6:**
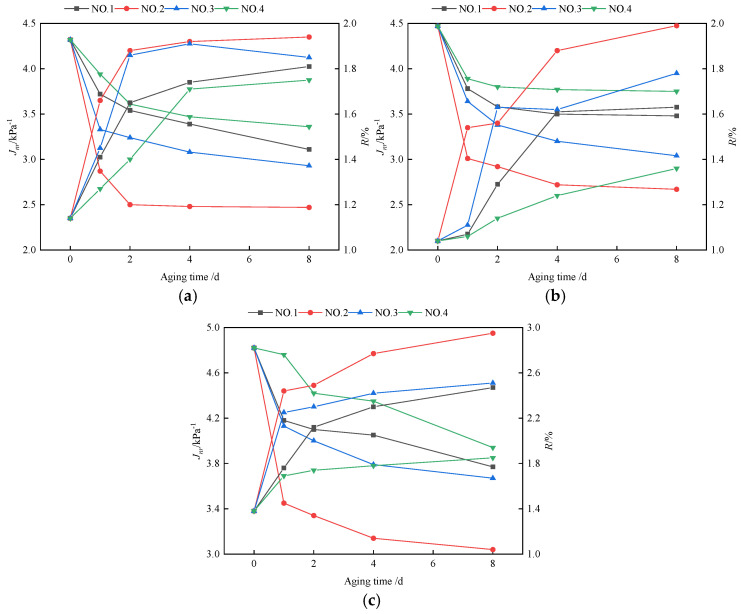
*J_nr_* and *R* changes in asphalt: (**a**) *J_nr_* and *R* changes in ZSY; (**b**) *J_nr_* and *R* changes in HF; (**c**) *J_nr_* and *R* changes in K.

In order to evaluate the influence of light–heat–oxygen coupling aging on the deformation and resilience properties of asphalt, the non-recoverable flexibility (*J_nr_*) and recovery rate (*R*) of the samples were determined. Since the changes in Jnr and R at 3.2 kPa are more representative compared to 0.1 kPa, the changes in *J_nr_* and *R* at a pressure of 3.2 kPa were selected. The results are shown in Figure 6.

It can be obtained from Figure 6 that with the increase in aging time, the Jnr value of asphalt gradually decreases, and the R value gradually increases, indicating that as the aging degree continuously increases, the ability of asphalt to resist high-temperature deformation and elastic recovery is enhanced. The reason is that as the degree of aging gradually increases, the components in the asphalt change. After the four types of base asphalt underwent ultraviolet aging, the content of light components decreased while the content of asphaltenes increased. Since the light components in asphalt mainly contribute to softening and lubricating, and the asphaltenes mainly act as the “skeleton”—that is, as the degree of aging increases—the components in the base asphalt that contribute to softening and lubricating decrease, while the components that contribute to the “skeleton” increase, eventually causing the base asphalt to develop towards an elastomer [22].

#### 3.2.2. The Change in Low-Temperature Rheological Properties Index

**Figure 7:**
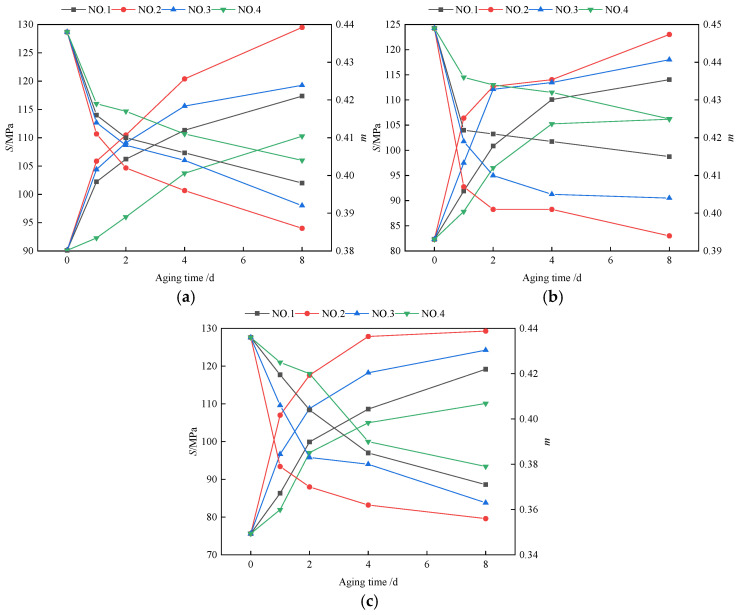
*S* and *m* changes in asphalt: (**a**) *S* and *m* changes in ZSY; (**b**) *S* and *m* changes in HF; (**c**) *S* and *m* changes in K.

The stiffness modulus (*S*) and creep rate (*m*) were used to evaluate the crack resistance and stress relaxation ability of asphalt in low-temperature environments, and the results are shown in Figure 7.

It can be seen from Figure 7 that with the increase in aging time, the *S* of asphalt gradually increases and the *m* gradually decreases, indicating that after aging, the low-temperature toughness and crack resistance of asphalt as a viscoelastic body gradually decline, and the low-temperature mechanical properties of asphalt are damaged. It can be seen from the figure that both *S* and *m* show a significant change trend in the early stage of aging and a gradual slowdown in the later stage, indicating that as the aging reaction gradually increases, the aging inside the asphalt is basically saturated, and the low-temperature performance essentially remains unchanged.

By comparing the *S* and *m* of asphalt in different experimental groups, it can be seen that compared with other experimental groups, the *S* of experimental group 2 is the largest, and the *m* is the smallest, indicating that aging under ultraviolet irradiation and high temperature causes the greatest damage to the low-temperature rheological properties of asphalt. Especially compared with experimental group 3 undergoing strong ultraviolet irradiation, significant changes can also be seen. This not only indicates that the intensification of ultraviolet irradiation in a single direction has a smaller effect on asphalt aging compared to an increase in the overall environmental temperature, but it also shows that there is a blocking effect on aging in the direction of ultraviolet light irradiation. When asphalt is exposed to ultraviolet light, the surface layer ages first, and the aging process gradually progresses into the deeper layers through molecular movement and surface cracking under long-term ultraviolet exposure. However, experiments have found that long-term aging of asphalt will form a carbonized film on the surface layer. The formation of the carbonized film not only indicates the saturation of surface asphalt aging but also has a reflective and blocking effect on ultraviolet light, thereby restricting the further development of ultraviolet aging into the deeper layers of asphalt [23]. Compared with other experimental groups, experimental group 4 exhibited the smallest change, proving that the essence of asphalt aging is oxidation, which is consistent with the results of the elemental composition test.

### 3.3. Correlation Analyses of Chemical Composition Index and Rheological Properties Index

Since the rheological properties of asphalt at high and low temperatures after aging are affected by its internal chemical composition, the gray correlation method was selected to calculate the correlation between the two and analyze the microscopic composition mechanism behind the macroscopic properties of asphalt aging. From the aforementioned analysis, it can be known that the overall change trends of the different types of asphalt and the experimental group are similar. Therefore, asphalt aged for 8 days was adopted here for the correlation calculation. Taking the rheological performance indicators *J_nr_, R, S*, and *m* of asphalt aging as the reference sequence, X_A_ to X_D_ were defined. Taking the chemical composition indicators *I_c_, H/C, SI*, and *CI* as the comparison sequence, X_1_ to X_4_ were defined. The results are shown in Table 5.

It can be seen from Table 4 that the high-temperature rheological performance indicators *J_nr_* and *R* of asphalt and the low-temperature rheological performance indicators *S* and *m* have relatively large correlation coefficients with the functional group composition indicators *SI* and *CI*, indicating that the changes in the rheological properties of asphalt are greatly affected by the changes in oxygen-containing functional groups.

Due to the aging effects of ultraviolet radiation and temperature, the broken-bond free small molecules of asphalt will combine with oxygen atoms in the air to form a large number of polar functional groups. The polar functional groups not only enhance the intermolecular forces within the asphalt because of their strong polarity, but also because they have a larger volume compared to non-polar functional groups. Therefore, it will increase the ratio of the molecular space of asphalt and reduce the active area of the internal molecules. The enhancement of intermolecular forces and the reduction in molecular activity areas will cause the molecular chains of asphalt to move with difficulty due to external forces and spatial reasons. At the same time, since the formation of polar functional groups requires the involvement of oxygen atoms, experimental analysis indicates that after oxygen atoms enter the interior of asphalt, they react with other elements due to their poor chemical stability and attach to the molecular main chain, thereby increasing the stiffness and molecular weight of the asphalt molecular main chain, making internal rotation of the molecular chain more difficult due to its own internal reasons.

The involvement of oxygen and the generation of polar functional groups hinder the activity of asphalt molecular chains from both internal and external aspects, thereby causing stress concentration due to reduced activity under stress, which, in turn, increases brittleness and cracking susceptibility while reducing low-temperature performance. The decline in molecular chain activity will also enhance the chemical stability of asphalt itself. Macroscopically, this is manifested as an increase in the hardness and consistency of asphalt, thereby strengthening its high-temperature rheological properties. From this, it can be known that the changes in the high- and low-temperature rheological properties of asphalt are all due to the presence of polar functional groups and oxygen. Therefore, polar functional groups and oxygen are the decisive factors affecting the performance of asphalt. At the same time, polar functional groups are formed by the internal elements of asphalt and oxygen atoms. Thus, the decisive environmental factor for the aging of asphalt is the oxygen content.

## 4. Discussion

This study systematically examines the coupled effects of light, heat, and oxygen on asphalt’s chemical and rheological evolution. Aging reduces aromatics and the *H/C* ratio, increases asphaltenes, and raises carbonyl and sulfoxide indices. These changes correlate with improved high-temperature performance but degraded low-temperature performance. Gray correlation analyses identified carbonyl and sulfoxide indices as the rheology-sensitive chemical markers, implying a causal link: polar oxygen-containing groups increase molecular rigidity and intermolecular forces, restricting chain mobility. The sulfoxide index shows higher sensitivity to aging, suggesting it may serve as an early-warning indicator of anti-aging capacity. Practically, accelerated aging protocols must ensure adequate oxygen supply, and anti-aging strategies should target suppression of polar functional groups. However, several limitations apply. The gray correlation analysis uses only day 8 (saturated) data, excluding earlier time points (0, 1, 2, and 4 days) to maximize signal-to-noise ratio; thus, the results reflect fully aged conditions only and do not capture transient early-stage correlations. Additionally, the 8-day period covers only early behavior; oxygen levels lacked intermediate gradients for dose response, and constant lab conditions cannot replicate field fluctuations. The use of 1.5 mm film and neat binder limits representativeness, and gray correlation does not prove causality. Future work should extend aging duration, incorporate more oxygen/environmental gradients, validate with field data, and extend to asphalt mixtures. These steps are essential to establish a realistic evaluation framework for durable asphalt design.

## 5. Conclusions

This paper analyzes the variation laws of macro and micro properties of asphalt under the coupling aging of light, heat, and oxygen in two contexts: the chemical composition mechanism and the decay law of rheological properties. On this basis, the relationship between the chemical composition and rheological properties of asphalt after aging is analyzed through correlation analysis. As such, the main research results are summarized as follows:

(1) Aromatic components existing in the form of long-chain molecules undergo bond-breaking and dehydrogenation reactions under the aging effect. The generated free small molecules are connected to aromatic rings, existing in the form of stable cyclic molecules, resulting in a decrease in aromatic component content and an increase in asphaltenes content, causing the asphalt to transform into a gel structure.

(2) After asphalt ages, dehydrogenation and oxidation reactions occur internally. Due to the entry of oxygen atoms, intermolecular forces are enhanced, increasing the stiffness of molecular chains. Therefore, it can be known that the essence of asphalt aging is oxidation.

(3) Free radicals in the molecule react with oxygen, causing the asphalt to produce C=O and S=O after aging. From the changes in *CI* and *SI* values, it was found that the carbonyl group can be used as an indicator to evaluate whether aging occurs, and the sulfoxide group can be used as an indicator for anti-aging ability.

(4) With an increase in aging time and degree, the *J_nr_* value of asphalt decreases, and the *R* value increases, indicating that the deformation and resilience properties increase. An increase in *S* and a decrease in *m* indicate a weakened ability to resist cracking and stress relaxation.

(5) Through correlation analysis, it was found that both the high- and low-temperature rheological properties of asphalt correlate significantly with the functional group index, and the oxygen content is a decisive environmental factor for asphalt aging.

This study provides microscopic theoretical and data support for understanding asphalt aging under coupled light–heat–oxygen conditions, which is of practical significance for the durability design of asphalt pavement in high-altitude, strong ultraviolet regions such as southwest China. However, several limitations should be acknowledged. The aging durations in this study may not capture long-term field aging behavior beyond this period. Only four environmental combinations were tested, and aging was conducted under laboratory conditions using a multi-functional environmental box, which cannot fully replicate the complex, fluctuating natural environment. Additionally, the oxygen content was set only at 0% and 20%, without intermediate levels. Future research should extend the aging time to cover longer equivalent service life, incorporate more gradient combinations of ultraviolet intensity, temperature, and oxygen content, and validate the laboratory findings with long-term field exposure data. Furthermore, developing anti-aging additives that specifically inhibit the formation of polar oxygen-containing functional groups represents a promising direction for improving asphalt durability.

## Figures and Tables

**Table 1:** Physical properties of asphalt.

Asphalt Sample	Penetration (25 °C, 0.1 mm)	Softening Point /°C	Elongation (15 °C)/cm	Density
ZSY	65.10	47.60	16.35	1.025
HF	76.70	46.05	48.00	1.036
K	74.30	48.60	23.40	1.030
Technical requirements	60~80	≥46	≥100	Actual measurement
Test method	T0604	T0606	T0605	T0603

**Table 2:** Ultraviolet irradiation intensity and monthly irradiation duration in each area.

Duration of Ultraviolet Irradiation Intensity	Region		
	The Four Southwestern Provinces	The Seventeen Eastern Provinces	The Nine Northwestern Provinces
Average irradiation intensity (W/m^2^)	21.51	26.68	32.00
Average monthly exposure duration (h)	116.00	200.00	266.00

**Table 3:** Light–heat–oxygen coupling aging scheme.

Experimental Grouping	UV Irradiation Intensity (W/m^2^)	Temperature (°C)	Oxygen Content (%)
Experimental group 1 (NO1)	65	40	20
Experimental group 2 (NO2)	65	60	20
Experimental group 3 (NO3)	85	40	20
Experimental group 4 (NO4)	65	40	0

NO1: 65 W/m^2^ UV + 40 °C + 20% O_2_ (normal temperature, normal oxygen, medium UV); NO2: 65 W/m^2^ UV + 60 °C + 20% O_2_ (high temperature, normal oxygen, medium UV; simulating the high temperature of the road surface in summer); NO3: 85 W/m^2^ UV + 40 °C + 20% O_2_ (strong UV, normal temperature, normal oxygen); NO4: 65 W/m^2^ UV + 40 °C + 0% O_2_ (oxygen-free control).

**Table 4:** Elemental composition change in asphalt.

Test Name	N Content (%)	C Content (%)	H Content (%)	S Content (%)	O Content (%)
ZSY–4–8	0.93	85.03	9.97	4.04	0.03
ZSY–4–4	0.97	84.95	10.02	4.05	0.02
ZSY–3–8	0.68	85.45	9.71	3.95	0.21
ZSY–3–4	0.69	85.35	9.75	4.00	0.21
ZSY–2–8	0.68	85.61	9.56	3.86	0.29
ZSY–2–-4	0.67	85.5	9.65	3.93	0.25
ZSY–1–8	0.69	85.31	9.76	4.00	0.25
ZSY–1–4	0.70	85.24	9.82	4.03	0.21
ZSY–0	0.73	84.97	10.13	4.06	0.12
HF–4–8	1.44	85.05	9.54	3.90	0.07
HF–4–4	1.40	84.58	9.81	4.19	0.02
HF–3–8	0.65	85.30	9.29	3.62	1.14
HF–3–4	0.68	85.01	9.52	3.85	0.95
HF–2–8	0.64	85.47	9.11	3.40	1.38
HF–2–4	0.67	85.04	9.42	3.73	1.13
HF–1–8	0.67	85.12	9.47	3.83	0.91
HF–1–4	0.68	84.98	9.61	3.97	0.76
HF–0	0.70	84.19	10.01	4.55	0.55
K–4–8	1.52	85.17	9.32	3.92	0.07
K–4–4	1.45	84.69	9.60	4.22	0.03
K–3–8	0.66	85.50	9.08	3.62	1.15
K–3–4	0.67	85.15	9.31	3.87	0.99
K–2–8	0.64	85.78	8.91	3.37	1.29
K–2–4	0.67	85.19	9.22	3.81	1.12
K–1–8	0.67	85.25	9.24	3.86	0.98
K–1–4	0.68	85.11	9.41	3.98	0.83
K–0	0.70	84.27	9.80	4.54	0.69

**Table 5:** Results of the correlation calculation.

Index	X_A_	X_B_	X_C_	X_D_
X_1_	0.6030	0.6076	0.6506	0.6410
X_2_	0.6297	0.6396	0.6561	0.6470
X_3_	0.8520	0.7920	0.8670	0.8490
X_4_	0.8890	0.8000	0.7426	0.7248

## Data Availability

The original contributions presented in this study are included in the article. Further inquiries can be directed to the corresponding author.

## Abbreviations

The following abbreviations are used in this manuscript:

Symbol	Description
*A_r_*	Aromatic content
*A_s_*	Asphaltene content
*R*	Resin content
*S*	Saturate content
*I_c_*	Colloidal instability index
*H/C*	Hydrogen-to-carbon ratio
*CI*	Carbonyl index
*SI*	Sulfoxide index
*J_nr_*	Non-recoverable creep compliance
*R*	Creep recovery rate
*S*	Creep stiffness modulus
*m*	Creep rate
MSCR	Multiple stress creep recovery test
BBR	Bending beam rheometer
UV	Ultraviolet

## References

[1] Wu M., Yin L., Li M., You Z., Jin D., Xin K. (2025). A state-of-the-art review of asphalt aging behavior at macro, micro, and molecular scales. Constr. Build. Mater..

[2] Office J.E., Chen J., Dan H., Ding Y., Gao Y., Guo M., Guo S., Han B., Hong B., Hou Y. (2021). New innovations in pavement materials and engineering: A review on pavement engineering research 2021. J. Traffic Transp. Eng. Engl. Ed..

[3] Kong L., Xi H., Peng Y., Zhu S. (2024). Review on UV Aging and Anti-Aging Materials of Road Asphalt. Mater. Rev..

[4] Camargo I.G.D.N., Hofko B., Mirwald J., Grothe H. (2020). Effect of Thermal and Oxidative Aging on Asphalt Binders Rheology and Chemical Composition. Materials.

[5] Zhu C. (2015). Evaluation of Thermal Oxidative Aging Effect on the Rheological Performance of Modified Asphalt Binders. Master’s Thesis.

[6] Zhu S., Qin X., Liao M., Ma Y., Xu H., Chen J., Gao H. (2023). Thermal Aging Degradation of High-Viscosity Asphalt Based on Rheological Methods. Materials.

[7] Hu Z., Xu T., Liu P., Oeser M., Wang H. (2020). Improvements of Developed Graphite Based Composite Anti-Aging Agent on Thermal Aging Properties of Asphalt. Materials.

[8] Pandhawale S.S., Jain S., Chandrappa A.K., Kari V. (2024). UV aging assessment of asphalt binder: Influence of duration, zinc oxide, and aging condition. Int. J. Pavement Eng..

[9] He Z., Geng J., Zhao W., Liu J., Qi C., Qi R., Huang L. (2025). Road performance and chemical evolution of aged asphalt subjected to intense ultraviolet radiation and large temperature fluctuations. Fuel.

[10] Gao M., Fan C., Chen X., Li M. (2022). Study on Ultraviolet Aging Performance of Composite Modified Asphalt Based on Rheological Properties and Molecular Dynamics Simulation. Adv. Mater. Sci. Eng..

[11] Li L., Huang C., Zeng X., Yang J., Gao W., Chen Y., Yang H. (2024). Rheological Performance and UV Aging Resistance of Buton Rock Asphalt and Styrene-Butadiene-Styrene Composite Modified Asphalt Under High Temperature. Coatings.

[12] Ye F., Sun D.Q., Huang P., Zhu J.P., Zhou Z.D. (2006). Analysis of Asphalt Photooxidation Aging Property Under Intensive Ultraviolet. China J. Highw. Transp..

[13] Liu H., Zhang Z., Tian Z., Li N., Li H., Wang P. (2022). Optimization of the evaluation indexes for asphalt UV aging behaviors based on multi-scale characterization methods: From the evolution of their physical-chemical properties and microscope morphology. Constr. Build. Mater..

[14] Chibiryaev A.M., Kozhevnikov I.V., Martyanov O.N. (2019). Transformation of petroleum asphaltenes in supercritical alcohols—A tool to change H/C ratio and remove S and N atoms from refined products. Catal. Today.

[15] Li H., Tong P., Zhang X., Lin X., Li B. (2020). Influence of Ultraviolet and Oxygen Coupling Aging on Rheological Properties and Functional Group Index of Warm Mix Asphalt Binder. Materials.

[16] Nasr D., Babagoli R., Mazrouei M. (2022). Evaluation of rheological behavior of asphalt binder modified by recycled polyethylene wax and crumb rubber. Constr. Build. Mater..

[17] Wu X., Milani G., Kang A., Liu P. (2025). Study on the flexural creep stiffness of chopped basalt fiber reinforced asphalt using finite elements and mean field homogenization. Compos. Struct..

[18] Zhang R., Sias J.E., Dave E.V. (2020). Correlating Laboratory Conditioning with Field Aging for Asphalt using Rheological Parameters. Transp. Res. Rec..

[19] Zhang D., Zhang K., Zheng Y., Guo H., Lu J., Jiang H., Qian G., Liang B. (2025). Gray correlation analysis of SBS-modified bitumen using atomic force microscopy at different aging phases. Constr. Build. Mater..

[20] Gui X., Long X., Zhan W. (2021). Performance Prediction Method of Bridge Asphalt Pavement Material Based on Grey Theory. Proceedings of the 2020 IEEE International Conference on Industrial Application of Artificial Intelligence (IAAI), Harbin, China, 25–27 December 2020.

[21] Fang Y., Zhang Z., Yang J., Li X. (2022). Performance Characterization of Biorecycled Asphalt and Gray Correlation Analysis between Its Components and Macroproperties. J. Mater. Civ. Eng..

[22] Xi H., Kong L.Y. (2025). Molecular Composition Changes of UV-aged Asphalt and its Structure-Activity Relationship with Rheological Properties and Chemical Composition. Mater. Rev..

[23] Li Y., Feng J., Yang F., Wu S., Liu Q., Liu Z., Li C., Gu D., Chen A., Jin Y. (2021). Gradient aging behaviors of asphalt aged by ultraviolet lights with various intensities. Constr. Build. Mater..

